# In Vitro Framework to Assess the Anti-*Helicobacter pylori* Potential of Lactic Acid Bacteria Secretions as Alternatives to Antibiotics

**DOI:** 10.3390/ijms22115650

**Published:** 2021-05-26

**Authors:** Samantha A. Whiteside, Mahi M. Mohiuddin, Sargon Shlimon, Jaspreet Chahal, Chad W. MacPherson, Jana Jass, Thomas A. Tompkins, Carole Creuzenet

**Affiliations:** 1 Infectious Diseases Research Group, Department of Microbiology and Immunology, University of Western Ontario, London, ON N6A 5C1, Canada; swhites@uwo.ca (S.A.W.); mmohiud6@uwo.ca (M.M.M.); sshlimon@uwo.ca (S.S.); jchahal3@gmail.com (J.C.); Jana.jass@oru.se (J.J.); 2 Lallemand Health Solutions, Montreal, QC H4P 2R2, Canada; cmacpherson@lallemand.com (C.W.M.); tttompkins@lallemand.com (T.A.T.); 3 The Canadian Research and Development Centre for Probiotics, London Health Research Institute, London, ON N6A 4V2, Canada

**Keywords:** *Helicobacter pylori*, lactic acid bacteria, secretome, urease, inflammation, cytokines, gastric disease

## Abstract

*Helicobacter pylori* is a prevalent bacterium that can cause gastric ulcers and cancers. Lactic acid bacteria (LAB) ameliorate treatment outcomes against *H. pylori,* suggesting that they could be a source of bioactive molecules usable as alternatives to current antibiotics for which resistance is mounting. We developed an in vitro framework to compare the anti-*H. pylori* properties of 25 LAB and their secretions against *H. pylori*. All studies were done at acidic and neutralized pH, with or without urea to mimic various gastric compartments. Eighteen LAB strains secreted molecules that curtailed the growth of *H. pylori* and the activity was urea-resistant in five LAB. Several LAB supernatants also reduced the urease activity of *H. pylori*. Pre-treatment of *H. pylori* with acidic LAB supernatants abrogated its flagella-mediated motility and decreased its ability to elicit pro-inflammatory IL-8 cytokine from human gastric cells, without reverting the *H. pylori*-induced repression of other pro-inflammatory cytokines. This study identified the LAB that have the most anti-*H. pylori* effects, decreasing its viability, its production of virulence factors, its motility and/or its ability to elicit pro-inflammatory IL-8 from gastric cells. Once identified, these molecules can be used as alternatives or complements to current antibiotics to fight *H. pylori* infections.

## 1. Introduction

*Helicobacter pylori* is a highly prevalent human gastric pathogen that globally infects ~50% of the world’s population and causes gastric ulcers and gastric cancers in 1–3% of infected individuals [1,2]. It is one of the few bacteria for which direct causality with cancer has been clearly established. Indeed, 1–3% of *H. pylori*-infected patients develop gastric cancer, 60–90% of gastric cancers are linked to *H. pylori* infection [3,4] and the risk of developing gastric cancer increases ~6-fold following infection with *H. pylori* [5,6]. *H. pylori* infections are typically chronic and accompanied by a high level of tissue inflammation, which is considered a critical first step in the formation of ulcers and the development of gastric cancer [3]. Current treatments combine a proton pump inhibitor (PPI) with two to three antibiotics and/or bismuth depending on bismuth availability and drug resistance profiles of the infections [7]. To account for frequent resistance to clarithromycin, metronidazole and levofloxacin, bismuth quadruple therapy that combines bismuth, a PPI, amoxicillin and clarithromycin or metronidazole is now recommended as a first-line treatment. It replaces the traditional clarithromycin-based triple therapy (clarithromycin with either metronidazole or amoxicillin and PPI). A “concomitant 4-drug” non bismuth therapy can also be used when bismuth is not available, including a PPI and each of amoxicillin, clarithromycin and metronidazole. Second-line therapies are adjusted based on the resistance profile of the infection. These drug combinations have side effects and are often poorly tolerated by patients, significantly impacting patient compliance with their treatment and the resulting eradication rates. The emergence of antibiotic resistant strains of *H. pylori* thus threatens the efficacy of existing treatments and calls for the identification of new, cost-effective means to combat infections. Several studies indicate that eradication of *H. pylori* is warranted as a means to reduce the global incidence and burden of gastric cancer, but this is not cost-effective using the available therapeutics without selectively targeting at-risk populations [8,9]. Novel strategies could reduce the incidence and burden of *H. pylori-*induced diseases by interfering with mechanisms involved in survival in the gastric environment and in the pro-inflammatory response to infection.

Accordingly, considerable research has focused on the characterization of *H. pylori*’s virulence factors [10] with the goal to inhibit their function or synthesis. An alternative approach is to screen for existing molecules able to interfere with virulence factors to provide new means to combat infections. The secretions from lactic acid bacteria (LAB) represent a potential source of such novel anti-*H. pylori* molecules that could be used as alternatives to the current antibiotics whose resistance is mounting.

Indeed, many studies have suggested that LAB ingestion is a beneficial complement to the treatment of *H. pylori* infection [11,12,13,14] and several studies have attributed the effects to secreted LAB molecules [15,16,17]. However, these studies have been performed either in vitro or in vivo (mouse models or in humans) and are highly diverse in terms of LAB and *H. pylori* strains used, the regimen of administration and the outputs measured. As such, the literature lacks a systematic analysis that permits direct comparison of the anti-*H. pylori* properties of various LAB strains, preventing the selection of the most active strains from which to derive active molecules.

We have developed an in vitro framework that allows well controlled, large scale comparative studies that are impractical in vivo but are necessary to identify the most active LAB under various pH and various urea concentrations that mimic the urea-rich gastric environment. Using this framework, we compared the secretions from 25 LAB for their effects on the growth of *H. pylori* strains NCTC 11637 (human isolate) and SS1 (mouse-adapted), on their ability to produce urease, lipopolysaccharide and flagella, and on their motility. All measured parameters are essential contributors to *H. pylori* colonization and pathogenicity. We also examined whether the LAB secretions affect *H. pylori*’s ability to modulate the secretion of cytokines by gastric cells. The *H. pylori* isolates chosen are well characterized in terms of virulence factors, and we chose to encompass a human and a mouse-adapted isolate to reflect in vivo studies that indicated beneficial effects of LAB and that were performed in humans and in mouse models. The selected LAB encompassed a wide variety of species and strains (Table 1), including specimens that are commercially available as dietary supplements and are thus considered safe for the human gastrointestinal tract. For example, strains L22 and L25 have been extensively tested and there is no evidence of toxicity [18,19]. The data can be exploited to identify the active molecules that could be used as alternatives to the current antibiotics used for the treatment of *H. pylori* infections whose antibiotic resistance is mounting, and the framework can also be exploited to derive mechanistic data.

## 2. Results

### 2.1. Live LAB Decrease the Viability of H. pylori in an Overlay Assay

**Figure 1:**
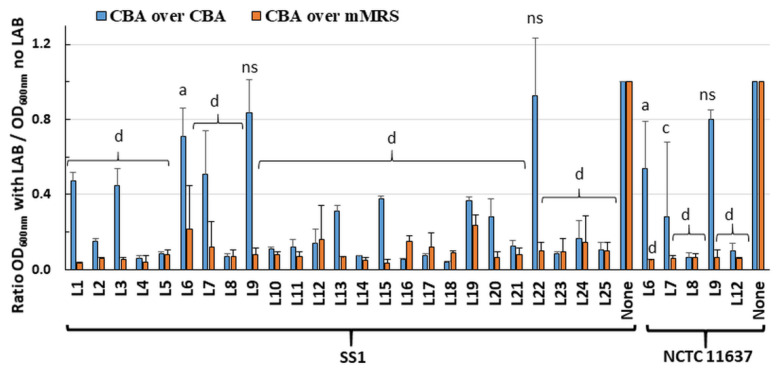
Anti-*H. pylori* activity of LAB in overlay assays. The LAB were grown embedded in the bottom layer (CBA or mMRS) of bi-layered plates. The top layer was always CBA. Afterwards, *H. pylori* was applied to the top layer and its growth was monitored after two days of incubation by harvesting the bacterial lawn and measuring the OD_600nm_ of the suspension. The data were normalized to the OD_600nm_ of the negative control that did not comprise any LAB; n = 2 to 5 biological replicates, with 1 technical replicate per biological replicate. Significance was assessed by one-way ANOVA with Dunnett’s multiple comparison test using the control without *H. pylori* and prepared in the same media as comparator; a: *p* < 0.05; c: *p* < 0.001; d: *p* < 0.0001; ns: not significant.

Twenty-five LAB strains (Table 1) were screened in an overlay assay for their ability to inhibit *H. pylori* growth. The plates comprised a bottom layer of Columbia blood agar (CBA) or modified deMan–Rogosa–Sharpe (mMRS) media to support growth of the embedded LAB, and a surface layer of CBA media to support *H. pylori* growth. Visual inspection indicated that most LAB grew to similar turbidity in CBA and mMRS. Using the CBA/CBA overlay, almost total inhibition of *H. pylori* strain SS1 growth was seen for nineteen of the twenty-five LAB (Figure 1). Using mMRS in the bottom layer greatly enhanced inhibition of *H. pylori* growth, with only 5–30% of residual growth compared to controls. Similar results were obtained for six LAB tested against *H. pylori* strain NCTC 11637 (Figure 1). Overall, these data suggest that most LAB secreted diffusible substances that inhibit the growth of *H. pylori*.

To rule out that these effects were only due to the LAB-mediated acidification of the media, the pH of the media was determined following the incubation of randomly selected LAB, including two strains (L21, L23) with killing ability in CBA/CBA media and three strains (L6, L9 and L22) devoid of activity. With the CBA/CBA combination, the pH of both layers remained at pH 7 notwithstanding the presence and growth of LAB. This indicated that diffusible molecules released by the LAB contribute to *H. pylori* growth inhibition in the absence of acidification. With the mMRS/CBA combination, the pH of both layers changed from pH 7 on control plates without LAB to pH 4.5 in the presence of LAB. The additional acid produced in the mMRS media may have contributed to the higher *H. pylori* growth inhibition observed with this media combination.

### 2.2. Most LAB Supernatants Harvested at 24 h Inhibit H. pylori Growth

Broth-based experiments were designed to provide a higher throughput in vitro framework to compare the effects of multiple LAB on *H. pylori* growth and virulence features under several well controlled conditions. In these assays, *H. pylori* was exposed for 48 h to spent filtered LAB supernatants, the pH of which was either neutralized or left at its native acidic value (Table S1). Potential pH effects were addressed by testing all LAB in the presence and absence of urea, which supports urease activity that is essential for survival of *H. pylori* in acidic conditions [24]. The effects of LAB supernatants on *H. pylori* growth were tested by colony forming unit (CFU) determination after drop-plating on LAB-free agar since preliminary experiments indicated that the LAB supernatants did not induce *H. pylori* lysis, preventing using OD_600nm_ values as viability or growth indicators (data not shown).

**Figure 2:**
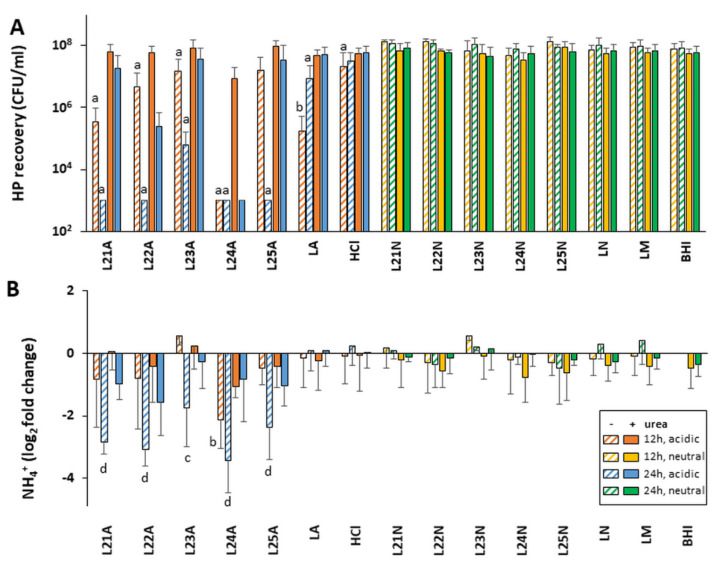
Effect of L21–L25 LAB supernatants on the growth and urease activity of *H. pylori* NCTC 11637. LAB supernatants from strains L21 to L25 were harvested after 12 h or 24 h of growth in mMRS broth under anaerobic conditions. *H. pylori* (HP) strain NCTC 11637 was exposed to 25% of these supernatants or control media without urea (hatched bars) or with 10 mM urea (solid bars) for 48 h. Growth was determined by spot plating and counting colony forming units (CFU) (**A**). The lower limit of detection for the assay was 10^3^ CFU/mL. The *H. pylori* cultures were also tested for the intracellular urease activity using the Berthelot reaction (**B**). The urease data are represented as the log_2_ fold change of the NH4^+^ produced by 180 µL of resuspended bacterial pellet over 30 min relative to the urea-free untreated control (BHI). The legend indicated in (**B**) applies to both panels. Specifically, the supernatants were left unmodified at their acidic pH (denoted A, blue and orange bars) or were neutralized to pH 7 by NaOH (denoted N, green and yellow bars). Controls included LAB growth media (LM), LM acidified to pH 4.5 by HCl (HCl), acidified to pH 4.5 by lactic acid (LA), or acidified to pH 4.5 by lactic acid then neutralized with NaOH (LN). As a positive control, *H. pylori* cultured in BHI without LAB supernatant or media were included. For each LAB, threeto five biological replicates comprising each threetechnical replicates were performed. For the controls, fiveto eightbiological replicates were performed. Significance was assessed by two-way ANOVA with Sidak multiple comparison test. BHI with matching time point and matching urea concentration was used as the comparator for each data set. Significance was represented as a, *p* < 0.05; b, *p* < 0.01; c, *p* < 0.001; and d, *p* < 0.0001. Lack of significance label indicates no significance.

Media controls incorporated in the assays were prepared with lactic acid (LA), which is relevant to LAB that produce LA, or with HCl to delineate if the nature of the acid present in the media (rather than only the pH) influences *H. pylori* growth. Of those two acid controls, only the LA control caused a slight decrease in *H. pylori* growth in absence of urea, and this was rescued by the presence of urea (Figure 2A). The LA control was used as comparator for LAB supernatants tested at acidic (native) pH. The LN control used to assess pH-neutralized LAB supernatants was prepared by first acidifying the media with LA (to mimic the presence of LAB) and then neutralizing with NaOH (LN). This did not affect *H. pylori* growth based on comparison with unaltered LAB growth media (LM) or *H. pylori* growth media (BHI).

**Figure 3:**
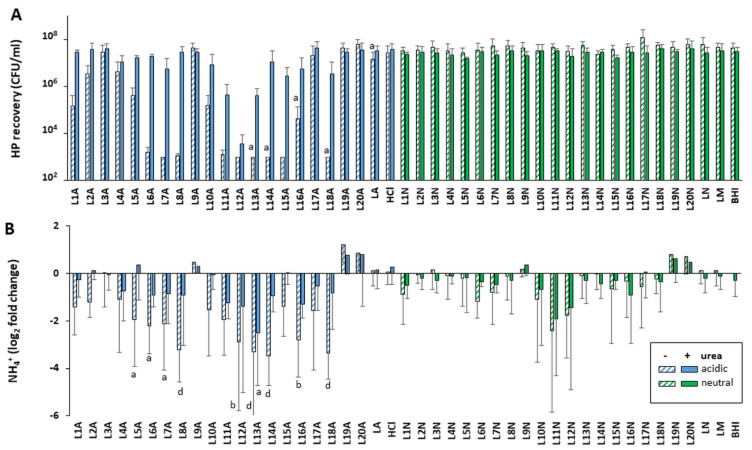
Effects of exposure to LAB L1–L20 supernatants on the viability and urease activity of *H. pylori* NCTC 11637. All LAB supernatants were harvested after 24 h of growth and *H. pylori* growth (**A**) and intracellular urease activity (**B**) were measured as described in Figure 2. Significance was represented as a, *p* < 0.05; b, *p* < 0.01; and d, *p* < 0.0001. Lack of significance label indicates no significance. For (**A**), three to four biological replicates were performed for each strain and ten to fifteen replicates of controls. For (**B**), four to five biological replicates were performed for each strain and eleven to fifteen replicates of controls.

Considering that bacterial metabolism, and therefore the secretion of active molecules, is tightly correlated with growth phase, the activity of LAB supernatants harvested in late exponential/early stationary phase (12 h) and late stationary phase (24 h) were first compared using a subset of LAB L21 to L25. These were chosen because they are already produced at industrial scale and commercialized for various probiotic applications. Their in-depth characterization is more likely to lead to prompt practical applications than others. Their secretions were not drastically different in terms of their pH, but as expected the culture density and bacterial viability decreased slightly in late stationary phase (Table S1). All five supernatants tested at acidic pH without urea showed very strong anti-*H. pylori* activity against both human NCTC 11637 isolate and mouse-adapted SS1 isolate when harvested at 24 h, with minimal activity for the matching 12 h native supernatants (Figure 2A and Figure S1A). The only exception was L24, which was active at both 12 h and 24 h. A higher level of activity was also observed with 24 h vs. 12 h native supernatants in the presence of urea. Thus, the screen of the remaining LAB supernatants L1 to L20 was conducted only with supernatants harvested at 24 h. This screen also identified several strains whose acidic supernatants could either totally abrogate or significantly reduce (2–3 logs) the growth of *H. pylori* when used in the absence of urea (Figure 3A and Figure S2A). A significant level of urea-resistant activity was also observed in several of these acidic LAB on at least one *H. pylori* strain. None of the 25 neutralized LAB supernatants harvested at 24 h demonstrated *H. pylori* growth inhibition, irrespective of the presence of urea.

Overall, L12 and L24 were the most potent of the 25 strains, abrogating the growth of both *H. pylori* NCTC 11637 and SS1 in the presence and absence of urea. Further, L24 demonstrated activity when harvested at both 12 and 24 h. Strains L11, L13 and L22 were also very efficient at curtailing *H. pylori* growth and showed significant urea resistance.

### 2.3. The Effects of LAB on H. pylori Growth Are Not Only Caused by Media Acidity 

**Figure 4:**
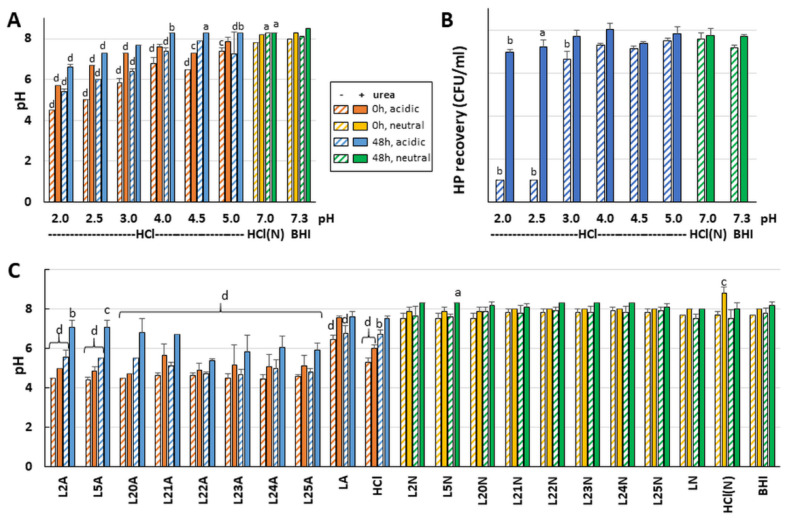
The activity of several LAB is due to bioactive molecules that exert their effects beyond pH effects. Exposure assays were carried out with media acidified with HCl in varying concentrations to generate a pH standard curve (**A**,**B**) or with LAB supernatants (**C**). The pH was monitored immediately after mixing each acidified media or LAB supernatant with *H. pylori* or after 48 h of exposure. The effect of HCl-mediated acidity on *H. pylori* recovery was recorded after 48 h of incubation (**B**). The effects of the LAB on *H. pylori* recovery were as presented in Figure 2 and Figure 3. The legend box applies to all panels. Specifically, samples with starting acidic pH are represented in orange (0 h) and blue (48 h) while samples with original neutral pH are represented in yellow (0 h) and green (48 h). Hatched bars signify the lack of urease while solid bars signify the presence of urea. Statistics were performed by one-way ANOVA with Dunnett’s multiple comparison test, using BHI of matching time and urea concentration as comparator. Significance was represented as a, *p* < 0.05; b, *p* < 0.01; c, *p* < 0.001; and d, *p* < 0.0001. Lack of significance label indicates no significance. For (**A**,**B**), two to three biological replicates were performed. For (**C**), three biological replicates were performed for each LAB strain, and four biological replicates were performed for the controls.

To tease apart the role of the media acidity from that of the LAB secretions on *H. pylori* growth, pH standard curves were built using HCl. Monitoring the pH evolution after incubation with *H. pylori* showed that the urease response was strong enough to neutralize the pH of the exposure mixture and to support *H. pylori* growth even without added urea as long as the starting pH was ≥5.5 (Figure 4A,B). Exogenous urea was only required to support *H. pylori* growth when the starting pH was ≤5, allowing the urease response to raise the pH to ~6 only.

The starting pH of exposure reactions made with the most active LAB strains was similar to the most acidic HCl control (for example, L11 and L12 in Table S1) but equally acidic starting pH were also observed for LAB that did not affect *H. pylori* growth (for example L4 and L20). This indicates that the acidic pH alone cannot account for the effects seen with active LAB and that LAB specific molecules exert an effect above and beyond that of the pH. To further confirm this, the starting and end point pH of exposure mixes was examined for a subset of LAB chosen for their range of effects on *H. pylori* growth (Figure 4C). The 4.5–5.0 starting pH did not increase after incubation with L22–L25 LAB supernatants without urea and only increased to 5.5–6.5 in the presence of urea. This mirrored the most acidic HCl control discussed above. While the rise to pH 6.0 was high enough to support growth of *H. pylori* in the HCl control, impaired growth was still observed for L22 and L24 (Figure 2). This indicates that these LAB comprise bioactive molecules affecting *H. pylori* growth beyond pH effects.

Monitoring the pH for L2, L5 and L20 that do not affect *H. pylori* growth under any condition further indicates that the pH is not the sole inhibitor of *H. pylori* growth by active LAB in absence of urea. Indeed, in exposures with L2, L5 and L20 in the absence of urea (Figure 4C), the pH remains acidic and equal to that observed for L22–L25, though without impairing *H. pylori* growth, while L22–L25 all impair growth in the same conditions. Hence, L22–L25 produce molecules that are active in the absence of urea beyond pH effects and are not present in L2, L5 or L20.

### 2.4. Native LAB Supernatants Decrease Urease Activity of Exposed H. pylori

Our pH and *H. pylori* recovery data above suggest a defect in the urease response after exposure to certain native LAB. To ascertain this, we measured the urease activity associated with *H. pylori* pellets recovered after 48 h of exposure to LAB supernatants. Untreated *H. pylori* grown in pH-neutral BHI harbored a significant amount of active urease despite the absence of any pH challenge, generating ~29 µmol NH_4_^+^/pellet of exposed *H. pylori* in a 30 min Berthelot assay. This basal amount of active urease was not changed upon addition of urea to the BHI control nor by variations in the control media composition or pH (Figure 2B and Figure 3B for NCTC 11637, and Figures S1B and S2B for SS1). It is thus sufficient to cope with moderate pH variations, including down to pH 5.5 seen in the HCl media control.

The basal urease activity was also not affected by exposure to neutralized LAB supernatants, irrespective of the presence of urea (Figure 2B and Figure 3B for NCTC 11637 and Figures S1B and S2B for SS1). However, it decreased 4–16 fold (−2 and −4 log_2_ fold change) following exposure to native LAB supernatants in the absence of urea, and up to 2fold in the presence of urea. While the strong urease activity reduction was seen with L21–L25 supernatants harvested at 24 h, no effect was seen with their 12 h counterparts that have similar pH, except for L24 (Figure 2B and Figure S1B). This pattern mirrors the effects of these supernatants on *H. pylori* growth, indicating that the LAB molecules affecting *H. pylori* growth also affect intracellular urease activity.

Overall, these data show that the amount of urease activity associated with LAB-exposed *H. pylori* pellets is dependent on the pH of the LAB supernatants (a decrease only seen with acidic supernatants) and the presence of urea (a decrease dampened with urea) and is affected negatively by molecules present in select LAB supernatants (mostly L21–L25). The LAB effects on urease activity supersede mere effects of the starting exposure pH since the starting pH in LAB L21–L25 was similar in the HCl control and in several LAB that show no urease activity reduction (for example L2, L20).

### 2.5. LAB Supernatants Do Not Affect Flagellin, Lipopolysaccharide or CagA Production but Affect Flagella-Mediated Motility

The Lewis Y lipopolysaccharide, CagA effector and flagellins are virulence factors essential for gastric colonization, host mimicry, chronic infection and/or inflammation. No differences in their production could be detected by Western blotting upon exposure to LAB L21–L25 supernatants under any conditions of pH and urea concentrations compared with controls (Figure S3). LAB L1–L20 also showed no defects for flagellin and lipopolysaccharide production when tested under neutral pH and without urea.

**Figure 5:**
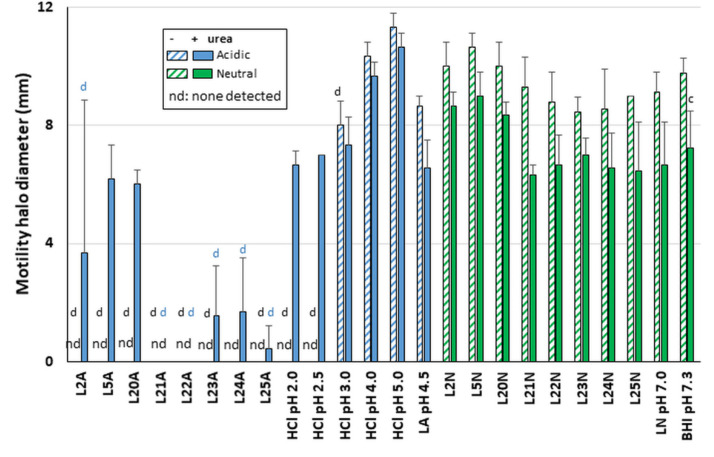
Effects of LAB supernatants and pH on *H. pylori* motility. *H. pylori* was stabbed in soft agar after 48 h of exposure to LAB or control, in the presence (solid bar) or absence (hatched bars) of urea. Samples with starting acidic pH (denoted A) are represented in blue while samples with starting neutral pH (denoted N) are represented in green. nd: none detected. Statistics performed as per Figure 2 relative to the BHI/no urea control. Statistics were performed by one-way ANOVA with Dunnett’s multiple comparison test, using BHI with matching urea concentration as comparator. Black letters for “BHI no urea” comparator and blue letters for “BHI + urea” comparator. Significance was represented as c, *p* < 0.001; and d, *p* < 0.0001 with two to three biological replicates per sample. Lack of significance label indicates no significance.

Since flagella-mediated motility is essential for gastric colonization, motility was also tested after stabbing exposure reactions in soft agar. Motility was preserved after exposure with all neutralized supernatants tested in LAB L21–L25, LAB L2, L5 and L20 (Figure 5). The data are consistent with our finding above that neutralized LAB supernatants do not affect flagellin production. In contrast, motility was abrogated after exposure to the acidic LAB supernatants in the absence of urea and was only rescued by the presence of urea for L2, L5 and L20. It remained abrogated or extremely limited for L21–L25 acidic supernatants even in the presence of urea. Full motility was observed in the acidic LA and HCl controls notwithstanding the presence of urea except under the most acidic conditions achieved with HCl (pH 2.0 and 2.5, Figure 5). Even in these cases, motility could be rescued by the presence of urea. Thus, the lack or limited motility seen with L21 to L25 in the presence of urea is specific to these LAB and is not simply caused by media acidity.

In all conditions where *H. pylori* was motile without urea, the presence of urea decreased the motility slightly. This could reflect the disruption of the flagella sheath’s integrity by chaotropic urea, which would result in acid-mediated and/or urea-mediated depolymerization of some of the flagellins, thus reducing motility. However, in vivo, the flagellar sheath is protective enough for *H. pylori* to swim from the pH 2–3 gastric lumen to the semi neutral gastric epithelium via the mucous layer, and thus acidity is not able to induce loss of flagellum structure or function despite the presence of gastric urea (the levels likely being too low to compromise the sheath integrity). Collectively, these data indicate that the lack of motility of acidic LAB even in absence of urea is not due solely to acid-mediated depolymerization of the flagellum but can be attributed to specific effects of LAB bioactive molecules that in some cases can be rescued by the presence of urea.

### 2.6. Global Cytokine Response Induced by Untreated H. pylori in AGS Cells

**Figure 6:**
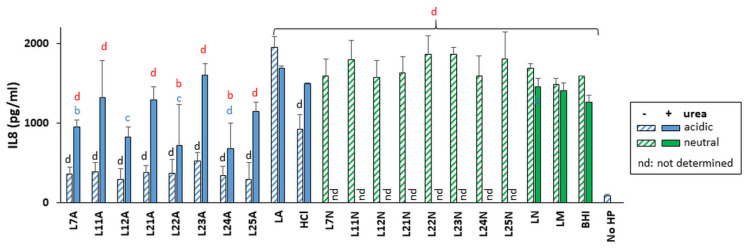
Effect of exposure of *H. pylori* to LAB supernatants on *H. pylori-*mediated secretion of IL-8 by gastric cells. IL-8 was measured in the supernatant of AGS cells exposed to *H. pylori* NCTC 11637 that had been pre-exposed to LAB supernatants or control media. The supernatants were left unmodified (acidic, A, blue bars) and added to *H. pylori* cultures without urea (hatched bars) or with 10 mM urea (solid bars); or were neutralized to pH 7 by NaOH (N, green bars) and added to *H. pylori* cultures without urea (hatched bars). Neutralized samples were not tested with urea, thus “nd” here indicates “not determined”. Controls included LAB growth media (LM), LM acidified to pH 4.5 by HCl (HCl), acidified to pH 4.5 by lactic acid (LA), or acidified to pH 4.5 by lactic acid then neutralized with NaOH (LN). As a positive control, *H. pylori* cultured in BHI without LAB supernatant or media control were included. All *H. pylori* controls were also completed in the presence or absence of 10 mM urea and followed the same color coding as test samples. Untreated AGS cell supernatant was included as a baseline (No HP). The data were derived from multiplex (27- and 40-plex Luminex assays) and ELISA experiments performed on the same samples, with three biological replicates in each experiment. The 27-plex and the ELISA data were normalized to the 40-plex data using the ratio of the average “urea-free, BHI” control values relative to the average “urea-free, BHI” control value of the 40-plex data. Statistics were performed by one-way ANOVA with Dunnett’s multiple comparison test. Significance was represented as b, *p* < 0.01; c, *p* < 0.001; and d, *p* < 0.0001. Lack of statistical label indicates no significance. Urea-free LA and LA containing 10 mM urea are the comparators for all acidic supernatants in the presence and absence of 10 mM of urea, depicted respectively in black and blue letters. Urea-free LN is the comparator for the pH adjusted (neutralized) supernatants in the absence of urea (no significance letters as none were significant). In addition, the “No HP” sample was used as a comparator to all test samples to demonstrate activation or lack thereof upon addition of *H. pylori* (red letters).

**Figure 7:**
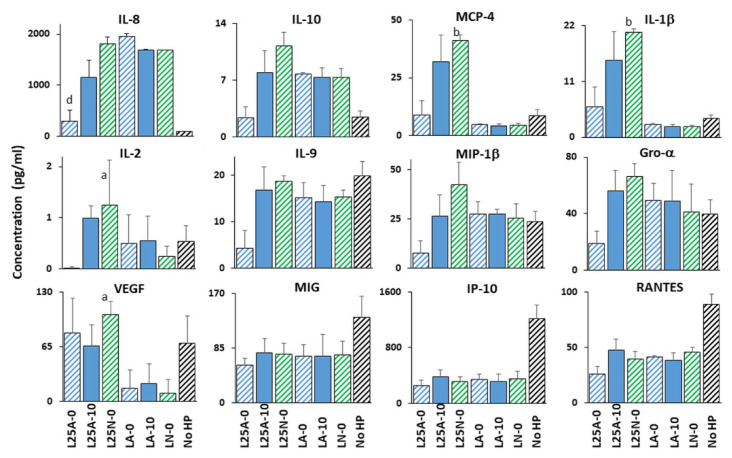
Effects of exposure of *H. pylori* to L25 supernatants on *H. pylori-*mediated secretion of cytokines by gastric cells. The data shown are extracted from Figure 6 and Figure S4 for NCTC 11637, with three biological replicates per sample. Only cytokines for which most differences were observed are shown, as well as 3 cytokines for which no effect occurred to demonstrate the specificity of the phenotypes. The supernatant left at its acidic pH (L25A) is compared to the LA media (hatched blue bars without urea; solid blue bars with 10 mM urea). The supernatant that was neutralized (L25N) is compared to the LN control media (hatched green bar, only determined in absence of urea). As a positive control, *H. pylori* cultured in BHI without LAB supernatant or media control were included (hatched black bars). Statistics were performed by 1-way ANOVA with Dunnett’s multiple comparison test. Significance was represented as a, *p* < 0.05 and b, *p* < 0.01. Urea-free LA and LA containing 10 mM urea are the comparators for all acidic supernatants in the presence and absence of 10 mM of urea, respectively. Urea-free LN is the comparator for the pH adjusted (neutralized) supernatants in the absence of urea. Lack of statistical label indicates no significance.

Upon interaction with *H. pylori*, gastric cells produce copious amounts of pro-inflammatory interleukin-8 (IL-8) [25,26]. Many other cytokines are also induced but most studies have examined a few cytokines at a time. To delineate the global cytokine response induced by *H. pylori* in gastric cells, a multiplex analysis was completed. It showed that the response of gastric cells to *H. pylori* NCTC 11637 grown under optimal conditions (no LAB challenge nor urea) is very specific and affects only 8 of the 54 cytokines tested. In particular, *H. pylori* increased the secretion of IL-8 15-fold (to 1500 pg/mL, Figure 6) and increased 2.2-fold the secretion of the anti-inflammatory cytokine interleukin-10 (IL-10, to 6 pg/mL) while decreasing 2- to 7-fold (starting from 150 pg/mL or less) the secretion of the pro-inflammatory cytokine interferon gamma-induced protein 10 (IP-10), monokine induced by gamma interferon (MIG), chemokine (C-C motif) ligand 5 (CCL5/RANTES), chemokine (C-X-C motif) ligand 16 (CXCL16/SCYB16) and the homodimer of platelet-derived growth factor subunit B (PDGF-BB) (Figure 7 and Table S2). There was also a trend towards decreased VEGF secretion, although this did not reach statistical significance.

### 2.7. Several LAB Supernatants Suppress H. pylori-Induced Secretion of IL8 by Gastric Cells

The ability of LAB supernatant-exposed *H. pylori* to induce an immune response was measured in gastric cells using eight LAB selected based on their *H. pylori* growth inhibitory properties, ranging from strong (L12 and L24) to moderate (L7, L11, L21, L22 and L25) and null (L23) in the presence of urea. *H. pylori* was pre-exposed to the supernatants and pelleted prior to transfer onto the gastric cells which were thus never in contact with the LAB supernatants. The data reflect the effects of LAB supernatants on the immunomodulatory properties of *H. pylori* towards gastric cells.

At their native pH and in the absence of urea, all eight LAB supernatants almost abrogated IL-8 secretion relative to the LA treatment (Figure 6). This dampening effect was lost or reduced in the presence of urea for most strains except L24, which also exhibited urea-resistance for most phenotypes described above. Pre-treatment of *H. pylori* with neutralized supernatants did not affect its ability to elicit IL-8 secretion from gastric cells.

The repression of RANTES, IP-10, MIG, PDGF-BB and SCYB16 seen with untreated *H. pylori* was maintained upon exposure of *H. pylori* to LAB supernatants (Figure 7 for L25 and Figure S4 for all other LAB and Table S2). In contrast, the *H. pylori*-mediated repression of VEGF was abolished in some cases. Further, pre-treatment of *H. pylori* with LAB supernatant did not alter cytokine secretion if the untreated *H. pylori* did not elicit a response, except for MCP-4, IL-1β and IL-2 which were induced by pre-exposure of *H. pylori* to neutralized supernatants of L25 and/or L22 in the absence of urea.

Of the eight LAB supernatants tested, L25 demonstrated the most global immunomodulatory power. It prevented the *H. pylori*-mediated repression of VEGF secretion, induced *H. pylori*-mediated downregulation of IL-8, IL-10, IL-9, MIP-1β and Gro α secretion at acidic pH in the absence of urea, and its neutralized supernatant induced *H. pylori*-mediated upregulation of MCP-4 and IL-1β in the absence of urea (Figure 7). These effects were cytokine specific and non-pleiotropic as L25 had no effect on *H. pylori’s* ability to repress IP-10, MIG or RANTES.

**Figure 8:**
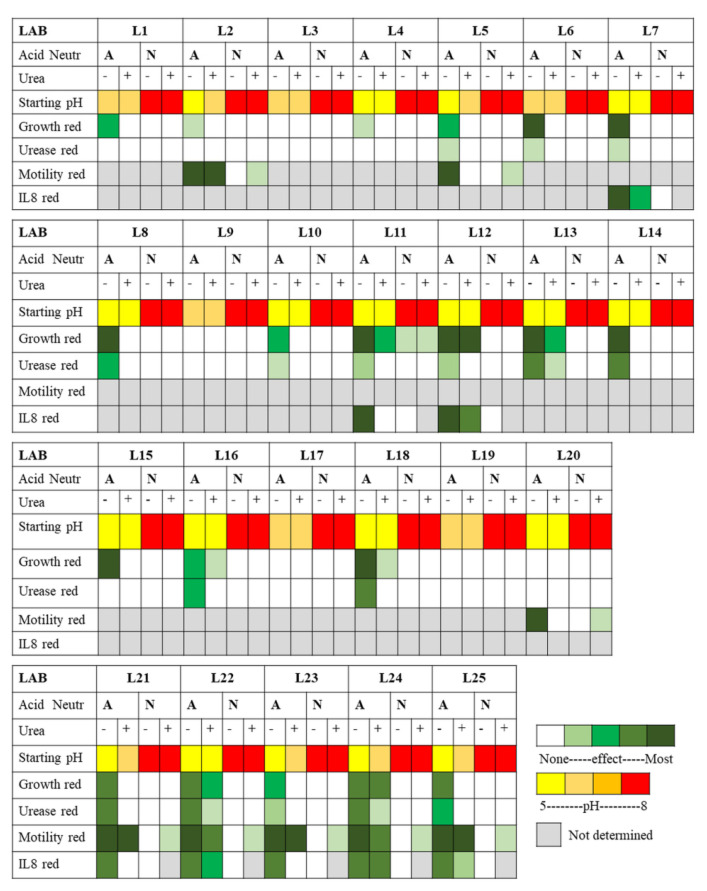
Heat map summary of all effects measured for the 25 LAB supernatants on *H. pylori* NCTC 11637. The supernatants were harvested at 24 h and used as their native acidic pH (A) or neutralized to pH 7 (N, Neutr). The starting pH of the exposure mixture is color coded from yellow (pH 4.5) to red (pH 8). For phenotypic effects, the color coding is relative to non-exposed *H. pylori* and depicts the reduction (red) of the original feature, from white for no effect to dark green for most effects. Grey indicates that no determination was performed.

The LAB-induced dampening of *H. pylori*-mediated inflammation is significant in itself and is also often combined with other beneficial LAB effects such as dampening of *H. pylori* growth and virulence features as summarized in Figure 8.

## 3. Discussion

### 3.1. Optimization of LAB Supernatant Exposure Assays

While overlay assays showed promising inhibition of *H. pylori* growth by various LAB, our in vitro broth-based framework enabled comparing the effects of secretions obtained from multiple LAB on *H. pylori* growth and virulence features. The various pH and urea combinations used are relevant to in vivo situations in the stomach. Gastric urea concentrations range from near 0 to 15 mM [27]. We used 10 mM urea, a concentration chosen based on our prior studies [28] and that does not cause any deleterious effects towards *H. pylori* growth or viability even when used in exposures with a neutral pH where urea is not needed for *H. pylori* survival. As for the pH, large variations occur as the bacteria travel from the acidic gastric lumen to the neutral epithelium via the gastric mucus. The studies performed using acidic supernatants mimic exposure in the stomach lumen, where LAB secretions may be used prophylactically to eliminate incoming *H. pylori*. However, once an infection is established, *H. pylori* resides underneath the gastric mucus in close proximity to the gastric epithelium where the local pH is close to neutral. As such, the studies done with neutralized supernatants are relevant to the use of LAB secretions for the eradication of established infections, whereby the active substances must retain their activity after neutralization. The addition of urea was also intended to provide proof of specific activity beyond pH effects as *H. pylori* utilizes urea from the stomach milieu to neutralize the local acidity [24].

In these assays, *H. pylori* was exposed for 48 h to spent filtered LAB supernatants. The 48 h time frame was chosen to account for the slow growth rate of *H. pylori* and for the need of sufficient time to convert any regulatory or transcriptional effects triggered by the LAB supernatants into functional and/or structural changes in *H. pylori*. For example, it allows for the turnover of potentially affected surface virulence features (such as lipopolysaccharide and flagellum) or the depletion of intracellular urease or CagA stores. The 48 h exposure also mimics the sustained presence of active molecules in patients medicated for at least 2 days. The time frame could not be extended beyond 48 h as *H. pylori* then takes on a coccoid shape. The coccoids are not culturable and represent a survival form rather than an active infectious form with well characterized virulence features.

### 3.2. Global Anti-H. pylori Effects of LAB Are Mediated by Secreted Molecules

**Figure 9:**
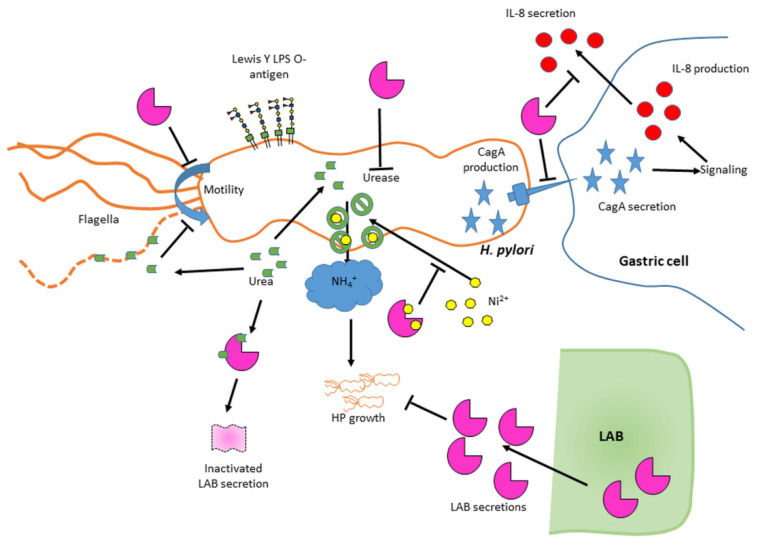
Model highlighting the various effects of LAB secretions on *H. pylori* virulence features. Activities vary across LAB and encompass direct effects on *H. pylori* growth, reduction of intracellular urease activity probably due to nickel chelation, abrogation of *H. pylori*’s ability to elicit IL-8 production likely via impairment of CagA secretion, and/or abrogation of motility. No effect was seen on flagellin, CagA or Lewis O-antigen production. Urea was able to inactivate some LAB secretions and to decrease *H. pylori* motility, likely via its chaotropic nature.

As summarized in Figure 8 and Figure 9, both our overlay and our broth-based supernatant assays show that specific LAB strains secrete molecules with anti-*H. pylori* effects against two *H. pylori* strains (human isolate NCTC 11637 and mouse-adapted SS1) as indicated by decreased growth, intracellular urease activity and motility after exposure, and also by the repressed induction of the pro-inflammatory response in gastric cells. These active LAB molecules are not produced in significant quantities until the late stationary growth phase and are only active if preserved at acidic pH. The different responses across the various LAB towards the same *H. pylori* strain suggest that the active molecules are different across LAB, a subset of strains secreting urea-resistant molecules (L11, L12, L13, L22 and especially L24) while others secrete highly urea-sensitive molecules.

### 3.3. The Anti-H. pylori Effects Are Mediated by Active Molecules beyond Lactic Acid and pH Effects

In addition to mimicking the stomach environment, urea was used to distinguish supernatants exerting a simple pH effect on *H. pylori* via their lactic acid production that acidifies the media from those that secreted active molecules. This was important since lactic acid contributes to the activity of LAB secretion against other pathogens [29,30] and the inhibition of *H. pylori* growth was most significant among the supernatants with pH < 4 with frequent loss of effect in the presence of urea. However, this loss did not correlate with the supernatant pH as five strains (L11, L12, L13, L22 and L24), which were among the most acidic, demonstrated urea-resistant *H. pylori* growth inhibition. We also observed that (i) the pH of the urea-sensitive supernatants was similar to that of strains that had urea-independent responses (L12 and L24; Table S1); (ii) the pH of the 12 and 24 h secretions was similar, yet the *H. pylori* inhibitory activity was drastically increased at 24 h; and (iii) there was limited, if any, growth inhibition in the acidic HCl and LA media controls in the absence of urea as long as the starting pH reached ~5.5.

Moreover, in agreement with prior studies [24,31], we observed that *H. pylori* produces enough urease to cope with the moderate pH decrease in LA and most HCl controls and that, while the addition of urea increased survival of *H. pylori* exposed to LA media, it was not essential as significant growth was still observed without urea. Thus, minimal levels of urea present in the media or in situ generation of urea [32,33] seem sufficient to provide basic protection, at least for all starting exposure pH ≥ 5.5. Finally, exogenous urea only increased growth by ~1–2 log in the LA media control but increased it by ~4–6 log in LAB supernatant exposures, suggesting the urea effect is not mediated only through urease activity. Collectively, this suggests that urea exerts chaotropic and dissociative effects on LAB secreted molecules in addition to being a urease substrate for pH control as depicted on Figure 9. Urea also appears as a double edge sword in the context of motility, slightly reducing motility via chaotropic activity under conditions where motility occurs without urea, but rescuing motility in acidic conditions where no motility could otherwise occur, very likely by virtue of supporting the urease response. This may explain why there is no direct correlation between motility and starting pH, urease activity or *H. pylori* growth.

### 3.4. LAB Molecules Interfere with Urease Function

Many native supernatants caused a strong decrease in the urease activity associated with *H. pylori* pellets after exposure, and this effect was dampened by the presence of urea and was not observed with neutralized supernatants nor media controls. There appears to be a correlation between the ability of these acidic LAB to suppress intracellular urease activity and to suppress neutralization of media acidity during exposure, whereby L21–L25 prevent *H. pylori* from neutralizing the media and reduce intracellular urease activity while L2, L5 and L20 do not impair pH neutralization nor intracellular urease activity. There also appears to be a correlation between the ability of acidic LAB to suppress intracellular urease activity and their effects on *H. pylori* growth, whereby strong decreases in urease activity are always associated with strong decreases in viability. However, decreased urease activity is not the only cause of reduced *H. pylori* growth since strong decreases in *H. pylori* growth also occurred in the absence of statistically significant effects on urease activity for supernatants that had the same low starting pH (for example, L6, L7, L11 and L15, Figure 4). In summary, in some LAB, the active molecules exert their effects on *H. pylori* growth in a urease- and pH-independent mode, while in others (including L21, L22, L24 and L25), the active LAB molecules affect the urease response, which in turn may impact *H. pylori* growth.

The decrease in intracellular urease activity did not result from cellular content leakage, as the unaffected OD_600nm_ suggests that *H. pylori* did not lyse. It also did not result from a limiting urea amount as additional urea is provided during the Berthelot assay. We propose that some LAB supernatants comprise molecule(s) that interfere with urease function rather than its expression, likely by preventing *H. pylori* from acquiring components necessary for urease activity, such as urea itself or nickel ions [34]. LAB are known to produce metal binding molecules [35,36]. Such molecules may be active in acidic conditions but their ability to chelate the nickel ions may be sensitive to the chaotropic nature of urea as depicted on Figure 9.

### 3.5. Multiplex Analysis Delineates the Cytokine Response of AGS Gastric Cells to H. pylori

Our cytokine multiplex data are consistent with other studies that described the induction of IL-8 secretion from gastric epithelial cells by *H. pylori* [37] and show that the cytokine response in the AGS cell system is rather narrow, only affecting 8 of the 54 cytokines tested. Many other cytokines are also linked to the pathogenesis of *H. pylori* including RANTES and IP-10 that are produced by tumor cells [38,39] and VEGF that increases in patients with gastric cancers and supports tumor growth via angiogenic activity [40]. However, in our study, we find them all repressed, suggesting that their production may be increased by other cell types in other studies.

Our work supports the position of IL-8 as the hallmark of *H. pylori* infection as the secretion of IL-8 was drastically increased compared to the smaller changes noted for the other cytokines. The small increase in anti-inflammatory IL-10, combined with the sizeable decreases in several pro-inflammatory cytokines, may allow *H. pylori* to fine tune the immune response, thereby avoiding an overt IL-8-mediated pro-inflammatory reaction that may lead to *H. pylori*’s elimination. This fine-tuned response may support *H. pylori’s* chronic establishment, which is characterized by chronic low-grade inflammation that causes limited, but continuous tissue damage from which nutrients can be derived.

### 3.6. LAB Secretions Protect against H. pylori-Induced Inflammation

Blocking the *H. pylori*-induced inflammation through the use of LAB secretions would be a significant milestone in decreasing the pathogenic impact of *H. pylori* infections. As seen with live LAB in other studies, the host response to LAB secretions is also species- and strain- specific. We observed several beneficial effects with most of the eight LAB supernatants tested, such as the repression of *H. pylori*-induced elicitation of pro-inflammatory IL8 and the maintenance of *H. pylori*-induced repression of pro-inflammatory IP10, MIG, VEGF, RANTES, and SCYB16 (Figure 7 and Figure 8 and Figure S4). The only two potentially deleterious effects noted were the increased secretion of pro-inflammatory IL-1β and MCP-4, which was the most pronounced with L25 and L22 while remaining at low levels (10–40 pg/mL). Their induction may not be sufficient to eliminate the beneficial curtailing of IL-8 secretion from 1500–1800 to ~500 pg/mL. Thus, the strains tested have the potential to curtail *H. pylori*-induced inflammation with no significant deleterious effects on the immune response of gastric cells. Given that inflammation promotes the development of gastric ulcers and cancers, the prevention of inflammation by LAB supernatants is of great clinical significance. Also, these effects are in line with recent studies that showed that the secretome of LAB L25 decreased the inflammatory response of human intestinal cells to Salmonella [41].

### 3.7. The Immunomodulatory Effects Likely Involve a Defect of CagA Transduction in Host Cells

CagA is considered the preponderant effector that stimulates IL-8 secretion after delivery inside host cells by live *H. pylori* [37]. Since we observed that the intracellular level of CagA was not affected by exposure to LAB supernatants, we surmise that its delivery to the host cell is compromised once *H. pylori* has been exposed to LAB supernatants (Figure 9). Decreased *H. pylori* viability would of course prevent CagA intracellular delivery and cause decreased IL-8 induction, but this is not the only factor at stake since reduction of IL-8 induction was noted with L23, which only partially reduced *H. pylori* growth, leaving ~10^5^ CFU/mL of live *H. pylori* for interactions with AGS cells. Thus, we propose that the LAB supernatants may comprise molecules that remain associated with *H. pylori* (surface-bound or internalized) and inhibit the Cag pilus formation and/or CagA secretion once *H. pylori* is transferred on the gastric cells.

### 3.8. Identified LAB with Most Anti-H. pylori Activity and Applications

Our study revealed that the secretions of many LAB can curtail *H. pylori* growth, reduce its motility, its intracellular urease activity and /or its ability to mediate inflammation, and that the performance is dependent on the LAB strain/species and on the conditions tested (pH, urea), as summarized in Figure 8 and Figure 9. This study allowed to delineate the most active LAB under gastric mimicking conditions (acidic and with urea) as L24, L12 and L13, all being *Lactiplantibacillus plantarum* isolates. All the *Lacticaseibacillus* *rhamnosus* isolates (L7, L8, L10, L11, L14, L15 and L25) inhibited *H. pylori* growth at native pH in absence of urea and most decreased urease activity and amongst them, L11 also showed very good urea resistance. The two *Limosilactobacillus reuteri* isolates, L5 and L6, demonstrated strong inhibition of *H. pylori* growth, yet no urea resistance. Overall, the most active strains were *L. plantarum* and *L.* *rhamnosus*, with a few very active isolates from *L.* *casei*, *L.* *paracasei*, *L.* *reuteri* and *L.* *helveticus* species. Of significance, both the L22 *L. helveticus* and L25 *L. rhamnosus* isolates improve chronic gastric inflammation in a mouse model of infection [42] though the contribution of their secretome to this phenomenon was never investigated.

The demonstrated activity of these supernatants against two largely unrelated *H. pylori* strains suggests that the secretions will be effective against a broad range of *H. pylori* strains, supporting their potential as prophylactic or therapeutic aids. Administration as a concentrated bolus of native lyophilized secretions when gastric conditions are acidic (outside mealtime) would allow maximal activity, allowing the secretions to diffuse through the gastric mucus to reach the epithelial surface and curtail the inflammatory capacity of established *H. pylori.* The activity would likely decay over time once the active molecules reach the neutral environment of the epithelium as indicated by the lack of activity observed for secretions stored after neutralization in our in vitro assays. However, we suspect that the decay may be slow enough to allow efficacy, as in vivo studies show an amelioration of symptoms and disease upon oral administration of LAB, indicating that their secretions are effective even on resident *H. pylori* established on the semi-neutral surface of the epithelium. The secretions could also prevent colonization by incoming *H. pylori* by inhibiting its acid survival, growth and flagella-mediated motility. While most active molecules appear sensitive to the chaotropic nature of urea, our screen revealed five strains with total (L12 and L24) or mostly (L11, L13 and L22) urea-resistant growth inhibition activity against *H. pylori*. Amongst these, L12, L22 and L24 also showed urea-resistant IL-8 repression. These strains thus have the greatest potential since urea resistance is a *sine qua non* condition for practical application in the stomach where urea is abundant.

## 4. Materials and Methods

### 4.1. Growth Conditions and Preparation of Cell-Free Culture Supernatants of LAB

The 25 LAB used are listed in Table 1. Freezer stocks were incubated overnight on deMan–Rogosa–Sharpe (MRS, Difco) agar plates at 37 °C under anaerobic conditions. Single colonies were inoculated into 5 mL of MRS broth, incubated overnight and the cultures were diluted 1/25 (*v*/*v*) in modified MRS broth. After 12 or 24 h, the OD_600nm_ was determined and the cultures were centrifuged at 4000× *g* for 15 min at 4 °C. The culture supernatants were either maintained at their native pH (~4.5) or adjusted to a neutral pH with 5 M NaOH, supernatants were filter-sterilized and stored at −80 °C. Modified MRS (mMRS) broth consists of 1% of protease peptone No. 3, 0.5% yeast extract, 2% glucose, 8.2 mM ammonium citrate, 60.9 mM sodium acetate, 0.4 mM magnesium sulfate, 0.3 mM manganese sulfate, 11 mM dipotassium phosphate, and 0.12 mM iron (II) sulfate. The modifications to the MRS broth were necessary to encourage maximum LAB growth without impacting *H. pylori* viability in subsequent steps. Commercially available MRS did not support the growth of *H. pylori.*

### 4.2. Growing H. pylori for Inhibition Experiments

*H. pylori* strains NCTC 11637 (ATCC 43504) [1,43] and SS1 [44] were used throughout these studies. Both were grown under microaerobic conditions (5% O_2_, 10% CO_2,_ 85% N_2_) at 37 °C unless indicated otherwise. To revive the bacteria, ~100 µL of freezer stock were deposited as a single drop on Columbia Blood Agar plates supplemented with 10 μg/mL vancomycin, 5 μg/mL amphotericin B, 5 μg/mL trimethoprim and 0.05% sodium pyruvate (CBA) and incubated for 48 h. The bacteria were then suspended in Brain Heart Infusion broth supplemented with 0.25% yeast extract (BHI-YE), the suspension was spread across a fresh CBA plate and incubated for 48 h.

### 4.3. Overlay Assays for Exposure of H. pylori to Embedded LAB

The LAB were prepared from freezer stocks as described above and incubated anaerobically on an MRS agar plate at 37 °C overnight. Afterwards, 5–7 colonies were harvested and inoculated into 10 mL MRS broth, which was incubated anaerobically at 37 °C overnight. The cultures were centrifuged for 30 min at 5000× *g* and the resulting pellet was resuspended in 25 mL phosphate buffered saline (PBS). This suspension was used to prepare the bottom layer of the overlay plates by mixing 5 mL suspension with 45 mL mMRS or CBA agar equilibrated at 60 °C; each petri dish received 10 mL of this mixture. A negative control was prepared using PBS without LAB. Once solidified, 10 mL CBA agar were added to create the top layer. This layer was also allowed to solidify prior to incubation under anaerobic conditions at 37 °C overnight.

Separately, *H. pylori* was cultured as above, harvested and resuspended in 0.85% saline at an OD_600nm_ of 0.2. The bacteria were then spread onto a CBA plate using a sterile cotton swab dipped in the suspension three times and incubated microaerobically at 37 °C for 48 h. The *H. pylori* bacteria were harvested and resuspended in BHI-YE at an OD_600nm_ of 0.5. This suspension was inoculated onto the overlay plates mentioned above (following the overnight anaerobic incubation for LAB growth), by spreading 100 µL of the *H. pylori* suspension using glass beads. The plate was incubated at 37 °C under microaerobic conditions for 48 h. The *H. pylori* grown on the surface were then recovered from the top layer using a cotton swab and resuspended in 1 mL BHI-YE broth. The OD_600nm_ of this suspension was measured, and the ratio between the OD_600nm_ of the LAB-containing plates and the control plates was determined, thus controlling internally for the growth of each *H. pylori* batch. Two to five independent experiments were performed per LAB strain, for each bottom layer media type and for each *H. pylori* strain with one measurement for each type of plate per biological replicate. The pH of both agar layers was recorded with pH indicator strips after the LAB incubation for three independent plates per stain and three areas per plate.

### 4.4. Exposure of H. pylori to LAB Supernatants

*H. pylori* was revived by two passages on CBA plates as described above and harvested in BHI-YE. The OD_600nm_ of the bacterial suspension was adjusted to 0.2 and the bacteria were spread across three CBA plates using a cotton swab dipped into the suspension and incubated for 48 h. The bacteria from all three plates were harvested and used to inoculate a single flask containing 25 mL BHI-YE supplemented with 10% fetal bovine serum, 10 μg/mL vancomycin, 5 μg/mL amphotericin B, 5 μg/mL trimethoprim and 0.05% sodium pyruvate (BHI) at an OD_600nm_ of 1.5. Broth cultures were incubated microaerobically for 48 h under agitation at 100 RPM. Following this incubation, the *H. pylori* broth cultures were centrifuged at 4500× *g* for 20 min and the pellet resuspended in fresh BHI at an OD_600nm_ of 1.7. The bacterial suspension was split in half, with one aliquot receiving urea at a final concentration of 10 mM. Exposures were performed in sterile 96-well plates by combining 150 μL *H. pylori* culture (+/−10 mM urea) with 50 μL LAB supernatant or media control. The plates were incubated microaerobically for 48 h under agitation at 100 RPM. Effects on *H. pylori* growth were assessed by drop plating serial 10-fold dilutions onto CBA and counting the colony forming units (CFU) after seven days of incubation. The remaining 180 μL of the culture was centrifuged for 20 min at 4500× *g* and 4 °C; the resulting pellet was stored at −80 °C for urease content determination. Experiments were completed in technical triplicates and biological triplicates at a minimum. As controls, mMRS broth was modified to pH 4.5 by HCl (HCl), to pH 4.5 by lactic acid (LA), or to pH 4.5 by lactic acid then adjusted to pH 7 by NaOH (LN). Unmodified mMRS broth (LM) and standard *H. pylori* growth media (BHI) were also included.

The pH of the exposure mixes was recorded at the time of exposure setup (0 h) and after the 48 h incubation using three-color pH indicator strips (pH 3–10, Sigma) by withdrawing aseptically 15 µL of the exposure reaction immediately after mixing exposure components or 48 h after incubation.

### 4.5. Motility Assays

*H. pylori* was stabbed in soft agar 0.4% agar in BHI-YE plates (supplemented with 10% FBS, 10 μg/mL vancomycin, 5 μg/mL amphotericin B, 5 μg/mL trimethoprim and 0.05% sodium pyruvate) after 48 h exposure and motility was recorded as the diameter of the motility halo after five days of incubation in microaerobic conditions.

### 4.6. Lipopolysaccharide, Flagellin and CagA Analyses

Whole cell pellets of *H. pylori* NCTC 11637 pre-exposed to LAB supernatants (1 mL total volume at OD_600nm_ 1.25) were solubilized in 200 µL of lysis buffer (2% SDS, 4% 2-mercaptoethanol, 10% glycerol, 1 M Tris pH 6.8 and bromophenol blue). The sample was boiled for 30 min and proteinase K was added to a final concentration of 0.5 µg/mL and the sample was further incubated for 1 h at 56 °C to digest all residual proteins [45]. The samples (20 µL) were then analyzed on 14% tricine gels (0.75 mm thick) and the lipopolysaccharide detected by silver staining [46]. Alternatively, pellets from 1.5 mL of exposure at OD_600nm_ 1 were solubilized in 112.5 µL of 0.625 M Tris pH 6.8, 2% SDS, 2% beta-mercaptoethanol, 10% glycerol and 0.002% bromophenol blue. Afterwards, 40 µL of the proteins were separated by SDS-PAGE on 15% gels of 1.5 mm thickness. After transfer to a nitrocellulose membrane, Ponceau S red was used to detect total proteins and immunoblotting was performed with anti-flagellin antibodies [28], commercial anti-Lewis Y (1/100 dilution, Abcam anti-Blood Group Lewis Y antibody [F3]) or anti-CagA antibodies (1/100 dilution, Santa Cruz Biotechnology). Secondary antibodies were used at 1/5000 dilution and were goat anti rabbit conjugated to Alexafluor 800 for flagellin blots, and goat anti mouse conjugated to Alexafluor 680 for CagA and Lewis Y blots.

### 4.7. Urease Activity Determination

A modification of the Berthelot reaction was used to monitor the ammonium ions derived from hydrolysis of urea by urease activity [47,48]. The *H. pylori* pellets from the LAB supernatant exposures were resuspended in 200 µL sodium phopshate buffer (10 mM, pH 7). The samples were diluted 1/10 in the same buffer and 10 µL of this suspension were added in duplicate to 90 µL of urease reaction buffer (2.5% sodium citrate and 0.5 mM urea in 5 mM sodium phosphate buffer, pH 7) in a 96-well plate. The plate was incubated at 25 °C for 30 min. Afterwards, the modified Berthelot method was performed to measure the ammonium ions formed based on the colorimetric reaction of the 100 µL sample above with 50 µL of 2-phenylphenol-nitroprusside reagent (3.22% 2-phenylphenol sodium salt tetrahydrate and 0.015% sodium nitroprusside), followed by 25 µL buffered sodium hypochlorite (1% sodium phosphate, 0.5% sodium hypochlorite, pH 13) and 100 µL water. The plate was incubated under gentle agitation in the dark at 25 °C for 30 min and the color was read at 670 nm. Standard curves were prepared using ammonium sulfate (range: 50–3500 µM). The urease activity is represented as the fold change in the measured concentration of NH_4_^+^ ions relative to the non-treated *H. pylori* control (BHI, 0 mM urea). A minimum of three biological replicates were performed with three technical replicates per *H. pylori* exposure and 2 technical replicates for the Berthelot reaction.

### 4.8. Tissue Culture of Gastric Cells and Their Exposure to H. pylori

These experiments were performed using human gastric epithelial cells (AGS cells, originally derived from female patient) and the human *H. pylori* isolate NCTC 11637. AGS cells were routinely maintained in F-12K Nutrient Mixture (Kaighn’s Modification) supplemented with 10% FBS and penicillin–streptomycin at 37 °C and 5% CO_2_. Cells were passed at 80–90% confluency. On the day the *H. pylori* were exposed to LAB secretions, the AGS cells were seeded at 2 × 10^5^ cells/mL in 96-well tissue-culture treated plates, which were incubated for 48 h. At the 48 h time point, the 96-well *H. pylori* exposure plate was centrifuged for 20 min at 4500× *g* and 25 °C. The resulting bacterial pellets were resuspended in 200 μL F-12K media supplemented with 10% FBS. The AGS cells were then washed with PBS, 150 μL of the LAB-exposed *H. pylori* and media control-exposed *H. pylori*, or tissue culture media alone were added to the AGS cell monolayer. The co-culture was incubated for 12 h at 37 °C and 5% CO_2_ and subsequently centrifuged for 15 min at 1000× *g* and 4 °C. The supernatant was transferred to a new 96-well plate and stored at -80 °C. *H. pylori* viability was confirmed by drop plating serial dilutions of the bacterial suspension to CBA and counting the CFU as indicated above. All media controls described above were included, in addition to the *H. pylori* negative control. Three independent experiments were conducted with one well, per condition, per experiment, except for positive and negative controls, which comprised of two to four wells per experiment. All LAB supernatants used for these experiments were harvested at 24 h.

### 4.9. Cytokine Measurements

Cytokines were measured using the Bio-Plex Pro 27-plex human cytokine and 40-plex human chemokine panels (Bio-Rad Laboratories, Hercules, CA, USA) according to the manufacturer’s instructions at Lallemand Health Solutions (Montreal, QC, Canada). As directed, standards were reconstituted in RPMI supplemented with 0.5% BSA and 50 µL of sample was added to the respective wells of the 96-well plates. The plates were read using a BioRad Bio-Plex 200 system, powered by Luminex xMAP technology. Fifty beads were read per well. Standard curves were generated using the Bio-Plex Manager software (v6.2), with an acceptable recovery range of 70–130%. Additionally, IL-8 was measured by ELISA using the “Ready-set-go” kit (eBioscience, San Diego, CA, USA) following the manufacturer’s instructions.

### 4.10. Statistical Analysis

Significance was assessed using GraphPad Prism (v8.3.0) by one-way ANOVA with Dunnett’s multiple comparison test or two-way ANOVA with Sidak multiple comparison test. The test and comparators used are specified in the legend to each figure. All statistical significance *p* values and repeat *n* values are provided in Tables S3–S9.

## Figures and Tables

**Table 1:** Strains of lactic acid bacteria used in the screen for potential probiotic effects on *Helicobacter pylori*.

Code	Bacteria ^1^	Origin	Reference
L1	*Limosilactobacillus fermentum* ATCC 11739 ^T^	Oral Swab	[20]
L2	*Limosilactobacillus fermentum* ATCC 23271	Human intestine	[21]
L3	*Lactobacillus johnsonii* DSM 20553	Sour milk	
L4	*Lacticaseibacillusparacasei* subsp *paracasei* ATCC 25302 ^T^	Unknown	
L5	*Limosilactobacillus reuteri* ATCC 23272 ^T^	Human intestine	
L6	*Limosilactobacillus reuteri* RC-14	Female urogenital tract	[21,22]
L7	*Lacticaseibacillus rhamnosus* ATCC 11443	Unknown	
L8	*Lacticaseibacillus rhamnosus* GR-1	Female urogenital tract	[21]
L9	*Lactobacillus gasseri* ATCC 33323 ^T^	Human gut	
L10	*Lacticaseibacillus rhamnosus* ATCC 7469 ^T^	Unknown	[21]
L11	*Lacticaseibacillus rhamnosus* GG ATCC 53103	Human intestine	
L12	*Lactiplantibacillus plantarum* ATCC 14917 ^T^	Pickled cabbage	[21]
L13	*Lactiplantibacillus plantarum* ATCC 10012	Unknown	
L14	*Lacticaseibacillus rhamnosus* NCFB 2964	Pleural fluid	[22]
L15	*Lacticaseibacillus rhamnosus* ATCC 27773	Derived from 7469 CmR	
L16	*Lactobacillus amylovorus* DSM 20552	Adult human intestine	
L17	*Lacticaseibacillus casei* ATCC393 ^T^	Dairy product, Cheese	[21]
L18	*Lacticaseibacillus casei* strain Shirota	Human intestine	[23]
L19	*Lactobacillus crispatus* ATCC 33820 ^T^	Eye	
L20	*Lactobacillus delbruecki* ATCC 9649	Sour grain mash	
L21	*Lacticaseibacillus casei* HA108	Dairy	LHS
L22	*Lactobacillus helveticus* R0052	Dairy Starter Culture	LHS
L23	*Lacticaseibacillus paracasei* HA196	Dairy	LHS
L24	*Lactiplantibacillusplantarum* R1012	Unknown	LHS
L25	*Lacticaseibacillus rhamnosus* R0011	Dairy	LHS

^1,^^T^ indicates type strain. ATCC—American type culture collection. DSM—Deutsche Sammlung von Microorganismen (German collection of Microorganisms and Cell cultures)—www.dsmz.de (accessed on 15 January 2021). NCFB—National Collection of Food Bacteria. LHS: Lallemand Health Solutions.

## Data Availability

All data included in this manuscript, none published separately.

## Supplementary Materials

The following are available online at https://www.mdpi.com/article/10.3390/ijms22115650/s1.
