# Supplementary material for: In Vitro Framework to Assess the Anti-Helicobacter pylori Potential of Lactic Acid Bacteria Secretions as Alternatives to Antibiotics
Source: Int J Mol Sci. 2021 May 26;22(11):5650. doi: 10.3390/ijms22115650 (PMC8198849; doi:10.3390/ijms22115650)
Supplement: Supplementary file 1 [file ijms-22-05650-s001.zip › Supplementary Figures/Captions for supplementary figures.docx]

**Legends to supplementary figures**

**Figure S1.** Effect of L21-L25 LAB supernatants on the viability and urease activity of *H. pylori* SS1. LAB supernatants from strains L21 to L25 were harvested after 12h or 24h of growth in mMRS broth under anaerobic conditions. *H. pylori* (HP) strain NCTC 11637 was exposed to 25% of these supernatants or control media without urea (hatched bars) or with 10 mM urea (solid bars) for 48h. Growth was determined by spot plating and counting colony forming units (CFU) (**A**). The lower limit of detection for the assay was 10^3^ CFU/ml. The *H. pylori* cultures were also tested for the intracellular urease activity using the Berthelot reaction (**B**). The urease data are represented as the log_2_ fold change of the NH4^+^ produced by 180 µl of resuspended bacterial pellet over 30 minutes relative to the urea-free untreated control (BHI). The legend indicated in (**B**) applies to both panels. Specifically, the supernatants were left unmodified at their acidic pH (denoted A, blue and orange bars) or were neutralized to pH 7.0 by NaOH (denoted N, green and yellow bars). Controls included LAB growth media (LM), LM acidified to pH 4.5 by HCl (HCl), acidified to pH 4.5 by lactic acid (LA), or acidified to pH 4.5 by lactic acid then neutralized with NaOH (LN). As a positive control, *H. pylori* cultured in BHI without LAB supernatant or media were included. For each sample 3 to 5 biological replicates comprising each three technical replicates were performed and 5 to 7 biological replicates were performed for the controls. Significance was assessed by 2-way ANOVA with Sidak multiple comparisons test. BHI with matching time point and matching urea concentration was used as the comparator for each data set, Significance was represented as a, p<0.05; b, p<0.01; c, p<0.001; and d, p<0.0001. Lack of significance label indicates no significance.

**Figure S2.** Effects of exposure to LAB L1-L20 supernatants on the viability and urease activity of *H. pylori* SS1. All LAB supernatants were harvested after 24h of growth, *H. pylori* viability (**A**) and intracellular urease activity (**B**) were measured as described in Figure S1.. For each sample 3 to 6 biological replicates comprising each three technical replicates were performed and 10 to 15 biological replicates were performed for the controls. Significance was assessed by 2-way ANOVA with Sidak multiple comparisons test. BHI with matching time point and matching urea concentration was used as the comparator for each data set, Significance was represented as a, p<0.05; b, p<0.01; c, p<0.001; and d, p<0.0001. Lack of significance label indicates no significance.

**Figure S3.** Effects of LAB supernatants on flagellin and lipopolysaccharide production in *H. pylori* NCTC 11637. (**A**) screen for L21-L25 in all conditions of pH and urea for flagellin A (FlaA) and CagA production by Western blot. (**B**) screen for L21-L25 for lipopolysaccharide Lewis Y O-antigen. Only the example of screen for acidic LAB supernatants in absence of urea is shown. Other conditions gave similar results. Panels C and D, representative examples shown for the screen of L1-L20 under neutral conditions and without urea for flagellins (Western blot, (**C**)) and for the O-antigen (O-Ag) and the Lipid A – core of the lipopolysaccharide detected by silver staining (**D**). On panel C, FlaA is overexpressed flagellin A as per Merkx-Jacques et al 2004. The flagellin appears as a doublet comprising FlaA and FlaB that cross-reacts with our anti-FlaA antibodies. For all panels, the Ponceau S red stain showing total protein loading amounts is included.

**Figure S4.** Effect of exposure of *H. pylori* to LAB supernatants on *H. pylori-*mediated secretion of 54 cytokines by gastric cells. All data were acquired using a 40-plex and a 27-plex screen, augmented by ELISA for IL8 only. Cytokines were measured in the supernatant of AGS cells exposed to *H. pylori* that had been pre-exposed to LAB supernatants or control media. The supernatants were left unmodified and added to *H. pylori* cultures without urea (blue hatched bars) or with 10 mM urea (solid blue bars); or were adjusted to pH 7.0 by NaOH and added to *H. pylori* cultures without urea (green hatched bars). Controls included LAB growth media (LM), LM acidified to pH 4.5 by HCl (HCl), acidified to pH 4.5 by lactic acid (LA), or acidified to pH 4.5 by lactic acid then neutralized with NaOH (LN). As a positive control, *H. pylori* cultured in BHI without LAB supernatant or media control were included. All *H. pylori* controls were completed in the presence (solid bars) or absence (hatched bars) of 10 mM urea. Untreated AGS cell supernatant was included as a baseline (no HP). The – and + signs refer to absence or presence of urea. LOQ indicates Limit of Quantitation defined by standard curve. The data is representative of three biological replicates. For statistics, urea-free SBY is used as a comparator for the “HP^-^” samples and all media controls. Urea-free LA and LA with 10 mM urea are the comparators for all native supernatants in the presence and absence of 10 mM of urea, respectively. Urea-free LN is the comparator for the pH adjusted (neutralized) supernatants in the absence of urea. Significance was determined using one-way ANOVA with Dunnett’s multiple comparisons-test. Significance is given as a, p<0.05; b, p<0.01; c, p<0.001; and d, p<0.0001.
