# Supplementary material for: In Vitro Framework to Assess the Anti-Helicobacter pylori Potential of Lactic Acid Bacteria Secretions as Alternatives to Antibiotics
Source: Int J Mol Sci. 2021 May 26;22(11):5650. doi: 10.3390/ijms22115650 (PMC8198849; doi:10.3390/ijms22115650)
Supplement: Supplementary file 1 [file ijms-22-05650-s001.zip › Supplementary Tables/Table S1 IJMS.docx]

**Table S1. Lactic acid bacteria growth properties in mMRS.**

| **Species and Strain** | **Study Code** | **12 hour (mean ± SD)** | | | **24 hour (mean ± SD)** | | |
| --- | --- | --- | --- | --- | --- | --- | --- |
|  |  | **pH** | **OD_600_** | **CFU** | **pH** | **OD_600_** | **CFU** |
| *Lactobacillus amylovorus* | | | | | | | |
| 20552 | L16 | 4.0 ± 0.2 | 1.3 ± 0.1 | 2.23 ± 1.64×10^9^ | 3.7 ± 0.1 | 1.5 ± 0.1 | 1.36 ± 0.97×10^9^ |
| *Lactobacillus crispatus* | | | | | | | |
| 33820 | L19 | 5.6 ± 0.1 | 0.1 ± 0.1 | 3.82 ± 3.57×10^7^ | 5.2 ± 0.5 | 0.2 ± 0.0 | 4.49 ± 3.02×10^7^ |
| *Lactobacillus delbrueckii* | | | | | | | |
| 9649 | L20 | 5.3 ± 0.1 | 0.2 ± 0.0 | 8.96 ± 8.41×10^7^ | 5.0 ± 0.3 | 0.2 ± 0.0 | 1.19 ± 1.07×10^8^ |
| *Lactobacillus gasseri* | | | | | | | |
| 33323 | L9 | 5.1 ± 0.1 | 5.4 ± 0.2 | 3.62 ± 2.59×10^7^ | 5.0 ± 0.1 | 0.2 ± 0.0 | 1.23 ± 1.94×10^9^ |
| *Lactobacillus helveticus* | | | | | | | |
| LHS - R0052 | L22 | 4.2 ± 0.2 | 1.0 ± 0.3 | 3.90 ± 0.90×10^8^ | 3.8 ± 0.2 | 1.2 ± 0.4 | 2.09 ± 0.53×10^8^ |
| *Lactobacillus johnsonii* | | | | | | | |
| 20553* | L3 | 5.9 | ND | 4.00 ×10^5^ | 4.9 ± 1.0 | 0.3 ± 0.3 | 7.20 ± 2.12×10^6^ |
| *Lacticaseibacillus casei* | | | | | | | |
| 393 | L17 | 5.1 ± 1.1 | 0.6 ± 0.8 | 1.47 ± 0.19×10^9^ | 4.3 ± 1.0 | 1.1 ± 0.7 | 1.39 ± 1.13×10^9^ |
| Shirota | L18 | 4.1 ± 0.1 | 1.2 ± 0.4 | 8.90 ± 6.51×10^8^ | 3.7 ± 0.1 | 1.5 ± 0.2 | 2.81 ± 3.35×10^9^ |
| LHS - HA108 | L21 | 4.0 ± 0.3 | 1.3 ± 0.2 | 8.23 ± 4.48×10^8^ | 3.8 ± 0.1 | 1.4 ± 0.2 | 6.00 ± 2.57×10^8^ |
| *Lacticaseibacillus paracasei* | | | | | | | |
| 25302* | L4 | 4.3 | 1.0 | 5.30 ×10^8^ | 3.7 ± 0.2 | 1.2 ± 0.2 | 7.35 ± 4.73×10^8^ |
| LHS - HA196 | L23 | 4.5 ± 0.5 | 0.9 ± 0.5 | 1.81 ± 1.09×10^9^ | 4.0 ± 0.20 | 1.2 ± 0.4 | 1.36 ± 0.67×10^9^ |
| *Lacticaseibacillus rhamnosus* | | | | | | | |
| 11443 | L7 | 4.1 ± 0.2 | 0.9 ± 0.3 | 1.38 ± 1.03×10^9^ | 3.7 ± 0.1 | 1.4 ± 0.1 | 5.46 ± 3.01×10^8^ |
| GR-1 | L8 | 4.1 ± 0.5 | 1.1 ± 0.2 | 4.33 ± 3.16×10^8^ | 3.9 ± 0.2 | 1.3 ± 0.2 | 3.60 ± 2.53×10^8^ |
| 7469 | L10 | 4.1 ± 0.4 | 0.8 ± 0.4 | 5.78 ± 6.01×10^8^ | 3.8 ± 0.3 | 1.3 ± 0.3 | 6.65 ± 1.87×10^8^ |
| 53103 | L11 | 4.0 ± 0.3 | 1.4 ± 0.1 | 4.24 ± 3.31×10^8^ | 3.7 ± 0.1 | 1.5 ± 0.1 | 2.96 ± 1.87×10^8^ |
| 2964 | L14 | 4.0 ± 0.2 | 1.3 ± 0.0 | 5.36 ± 3.66×10^8^ | 3.7 ± 0.1 | 1.4 ± 0.3 | 1.70 ± 0.86×10^8^ |
| 27773 | L15 | 4.4 ± 0.2 | 0.7 ± 0.3 | 8.70 ± 8.49×10^8^ | 3.9 ± 0.2 | 1.2 ± 0.0 | 1.00 ± 0.07×10^9^ |
| LHS - R0011 | L25 | 4.2 ± 0.4 | 1.2 ± 0.3 | 1.72 ± 1.65×10^9^ | 3.8 ± 0.2 | 1.4 ± 0.2 | 1.51 ± 1.02×10^9^ |
| *Lactiplantibacillus plantarum* | | | | | | | |
| 14917 | L12 | 4.0 ± 0.2 | 1.5 ± 0.1 | 1.74 ± 0.86×10^9^ | 3.7 ± 0.1 | 1.4 ± 0.2 | 1.19 ± 0.50×10^9^ |
| 10012 | L13 | 4.0 ± 0.2 | 1.2 ± 0.2 | 2.82 ± 1.83×10^9^ | 3.8 ± 0.1 | 1.3 ± 0.2 | 2.45 ± 1.39×10^9^ |
| LHS - R1012 | L24 | 3.8 ± 0.1 | 1.5 ± 0.2 | 2.40 ± 1.55×10^9^ | 3.7 ± 0.2 | 1.6 ± 0.1 | 2.05 ± 1.69×10^9^ |
| *Limosilactobacillus fermentum* | | | | | | | |
| 11739 | L1 | 4.3 ± 0.2 | 0.9 ± 0.3 | 0.84 ± 1.18×10^9^ | 4.2 ± 0.1 | 1.2 ± 0.2 | 2.82 ± 4.17×10^8^ |
| 23271 | L2 | 4.1 ± 0.1 | 1.1 ± 0.2 | 1.56 ± 0.80×10^9^ | 4.2 ± 0.1 | 1.1 ± 0.3 | 1.33 ± 1.23×10^9^ |
| *Limosilactobacillus reuteri* | | | | | | | |
| 23272 | L5 | 4.7 ± 0.9 | 0.6 ± 0.5 | 1.24 ± 1.39×10^9^ | 4.2 ± 0.1 | 1.1 ± 0.2 | 1.17 ± 1.45×10^9^ |
| RC-14 | L6 | 4.6 ± 0.7 | 0.7 ± 0.6 | 8.83 ± 8.05×10^8^ | 4.1 ± 0.1 | 1.0 ± 0.3 | 1.46 ± 0.69×10^9^ |

* 12-hour data was only collected from one biological replicate.
