# Supplementary figures and images for: In Vitro Framework to Assess the Anti-Helicobacter pylori Potential of Lactic Acid Bacteria Secretions as Alternatives to Antibiotics
Source: Int J Mol Sci. 2021 May 26;22(11):5650. doi: 10.3390/ijms22115650 (PMC8198849; doi:10.3390/ijms22115650)

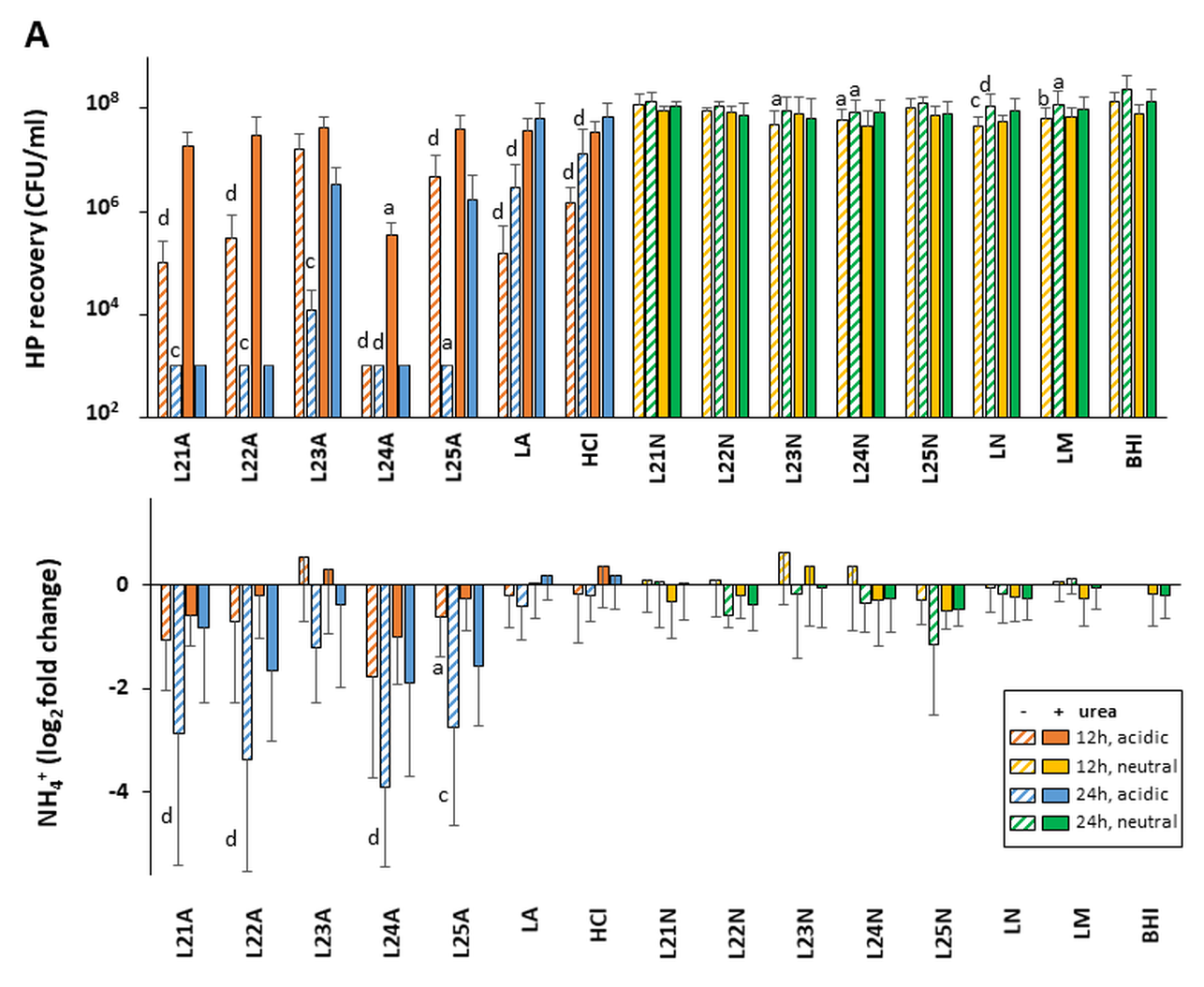

Supplement: Supplementary file 1 [file ijms-22-05650-s001.zip › Supplementary Figures/Figure S1_new.tif]

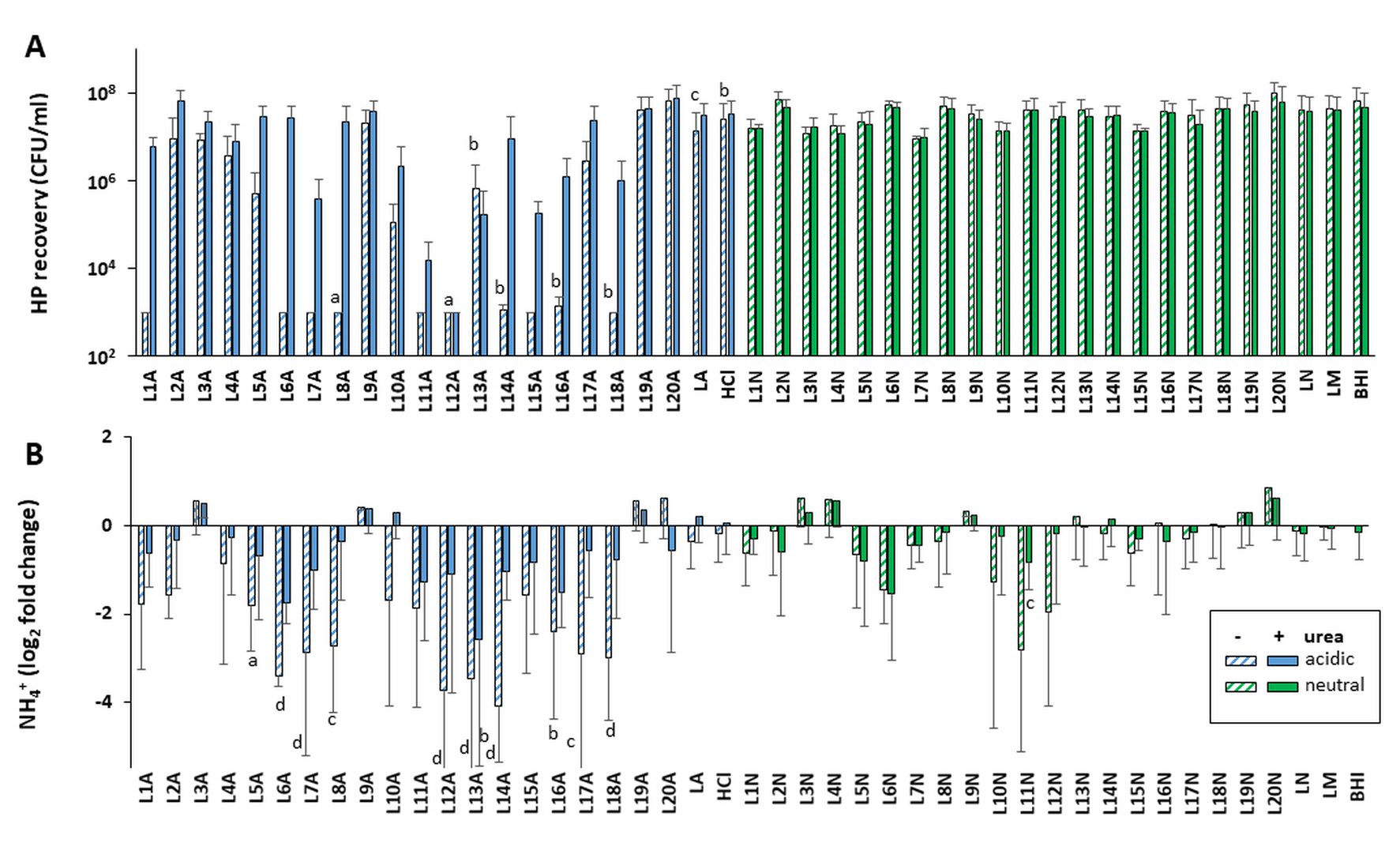

Supplement: Supplementary file 1 [file ijms-22-05650-s001.zip › Supplementary Figures/Figure S2_new.tif]

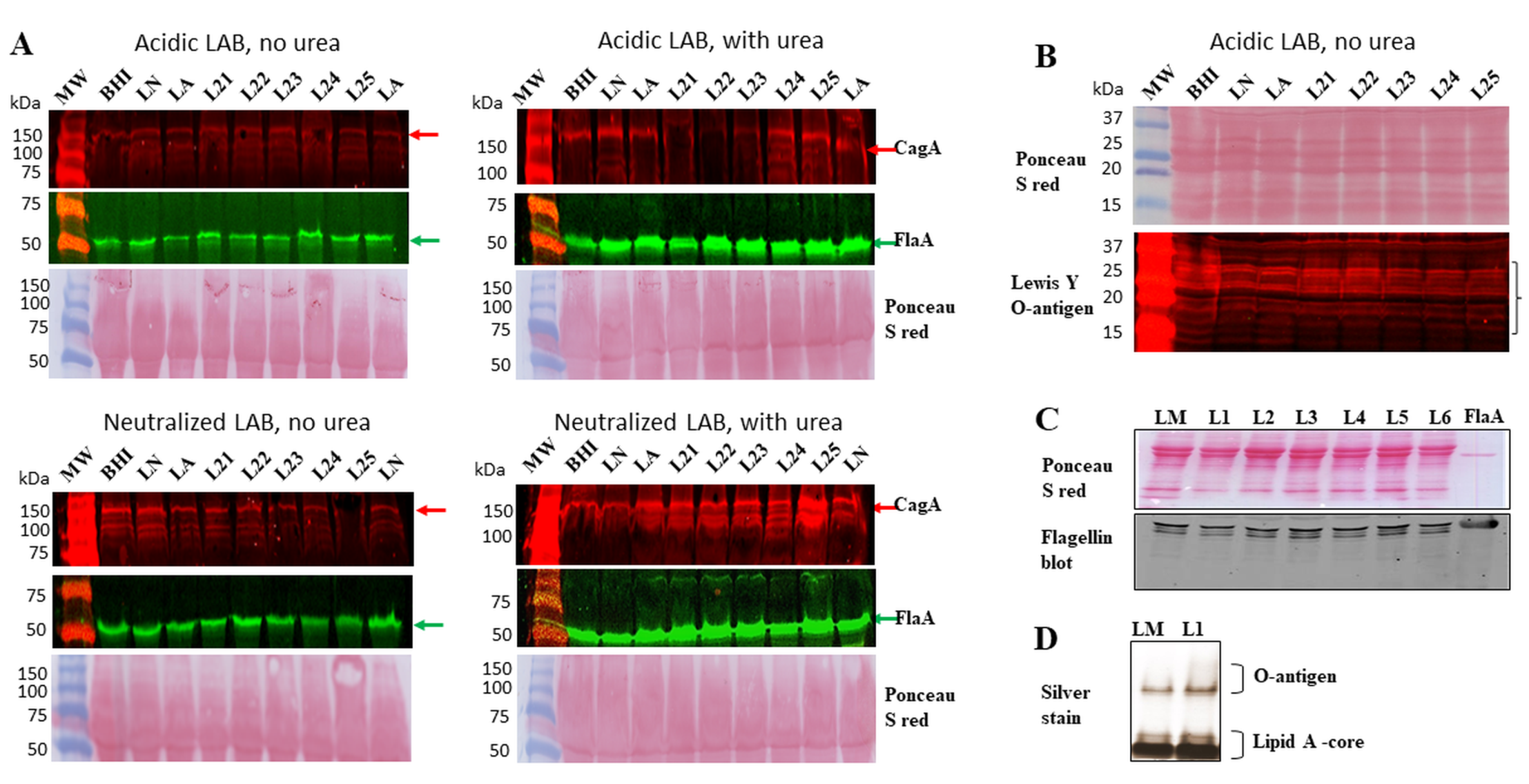

Supplement: Supplementary file 1 [file ijms-22-05650-s001.zip › Supplementary Figures/Figure S3.tif]
