# Supplementary material for: In Vitro Framework to Assess the Anti-Helicobacter pylori Potential of Lactic Acid Bacteria Secretions as Alternatives to Antibiotics
Source: Int J Mol Sci. 2021 May 26;22(11):5650. doi: 10.3390/ijms22115650 (PMC8198849; doi:10.3390/ijms22115650)
Supplement: Supplementary file 1 [file ijms-22-05650-s001.zip › Supplementary Tables/Table S2 IJMS.doc]

**Table S2: Cytokine and chemokine panel results.**

ns: not significant. ULOQ and LLOQ: upper and lower limits of quantitation. HP: *H. pylori*. LAB: lactic acid bacteria. Bold and colors highlight cytokines with most effects, yellow for decreases and grey for increases in cytokine level compared with relevant control.

| **Analyte** | **ULOQ (pg/ml)** | **LLOQ (pg/ml)** | **Level in AGS cells (pg/ml)** | **Effect of non-exposed HP on gastric cells** | **Effect of LAB pre-exposure of HP on HP’s ability to modulate cytokines in gastric cells** |
| --- | --- | --- | --- | --- | --- |
| 6Ckine | 41782 | 170.9 | 0 | No effect | No effect |
| Basic FGF | 16832 | 3.8 | 0 | No effect | No effect |
| BCA-1 | 6279 | 0.5 | 0 | No effect | No effect |
| CTACK | 19325 | 4.3 | LLOQ | No effect | No effect |
| ENA-78 | 195401 | 186.0 | 0 | Slight increase, ns | Increase with LAB in LA-10 and LN-0, ns |
| Eotaxin | 3384 | 6.2 | 0 | No effect | No effect |
| Eotaxin-2 | 22960 | 6.3 | 30 | No effect | No effect |
| Eotaxin-3 | 31929 | 28.4 | 0 | No effect | No effect |
| Fractalkine | 48842 | 11.9 | LLOQ | No effect | No effect |
| G-CSF | 108541 | 6.9 | 180 | No effect | No effect |
| GCP-2 | 13685 | 3.1 | 0 | No effect | No effect |
| GM-CSF | 34534 | 0.4 | 0 | No effect. | No effect |
| Gro-α | 13715 | 15.0 | 40 | No effect | No effect |
| Gro-β | 20422 | 18.8 | 0 | Slight increase, ns | No effect |
| I-309CCL1 | 8688 | 3.3 | 0 | No effect | No effect |
| I-TAC | 3327 | 0.7 | 50 | No effect | No effect |
| IFN-y | 30569 | 7.7 | 0 | No effect | No effect |
| **IL-1β** | **19412** | 2.3 | **0** | **No effect** | **Increase with 2 LAB** |
| IL-1RA | 38696 | 4.1 | 10 | No effect. | No effect |
| IL-2 | 41433 | 1.0 | 0 | No effect | Small increase for R0011 in LN 0 media |
| IL-4 | 27197 | 0.4 | 0 | No effect. | No effect |
| IL-5 | 20209 | 3.7 | 0 | No effect | No effect |
| IL-6 | 4983 | 0.7 | 0 | No effect | No effect |
| IL-7 | 146305 | 10.1 | 0 | No effect | No effect |
| **IL-8** | **35350** | 1.3 | **100** | **Increased 15-18-fold to 1500-1800 pg/ml** | **Dampened induction** |
| IL-9 | 27873 | 2.9 | 20 | No effect | No effect |
| **IL-10** | **90816** | **1.1** | **2** | **Increased to 6 pg/ml** | **LA-0 prevented induction but LN-0 enhanced induction** |
| IL-12 p70 | 9175 | 1.2 | 0 | No effect | No effect |
| IL-13 | 18971 | 0.3 | 0 | No effect | No effect |
| IL-15 | 1584 | 8.4 | 0 | No effect. | No effect |
| IL-16 | 61095 | 7.6 | 0 | No effect. | No effect |
| IL-17A | 11479 | 1.7 | LLOQ | No effect | No effect |
| **IP-10** | **51235** | 5.3 | **150** | **Decreased to ~25 pg/ml.** | **No change from HP only level** |
| MCP-1 | 7551 | 0.3 | 0 | No effect | No effect |
| MCP-2 | 4336 | 1.1 | 0 | No effect | No effect |
| MCP-3 | 44369 | 8.5 | 0 | No effect | No effect |
| **MCP-4** | **3572** | 16.0 | **<LLOQ** | **No effect** | **Increase from HP only level** |
| MDC | 5854 | 6.4 | 0 | No effect | No effect |
| MIF | 471187 | 109.9 | 1000 | No effect | No effect |
| **MIG** | **24091** | 4.6 | **140** | **Decreased to 75 pg/ml** | **No change from HP only level** |
| MIP-1α | 1401 | 1.4 | 20 | No effect | No effect |
| MIP-1β | 7771 | 0.5 | 25 | No effect | **LA-0 decreased to ~10 pg/ml** |
| MIP-1δ | 4920 | 1.6 | 35 | No effect | No effect |
| MIP-3α | 6981 | 1.0 | 0 | No effect | No effect |
| MIP-3β | 51523 | 10.0 | 0 | No effect | No effect |
| MPIF-1 | 19602 | 4.5 | 0 | No effect | No effect |
| **PDGF-BB** | **36953** | **14.3** | **55** | **Reduced to 30 pg/ml** | **No change from HP only level** |
| **RANTES** | **12056** | 4.2 | **100** | **Decreased to 40 pg/ml** | **No change from HP only level** |
| **SCYB16** | **7672** | 2.0 | **140** | **Decreased to 50 pg/ml** | **No change from HP only level** |
| SDF-1(α+β) | 38773 | 40.0 | 0 | No effect | No effect |
| TARC | 5416 | 25.3 | 0 | No effect | No effect |
| TECK / CCL25 | 116369 | 9.3 | 0 | No effect | No effect |
| TNF-α | 53670 | 3.5 | 0 | No effect | No effect |
| **VEGF** | **179321** | **50.4** | **70** | **Reduced to 20 pg/ml, ns** | **Trend toward reverting HP effect, ns** |
