# Supplementary material for: In Vitro Framework to Assess the Anti-Helicobacter pylori Potential of Lactic Acid Bacteria Secretions as Alternatives to Antibiotics
Source: Int J Mol Sci. 2021 May 26;22(11):5650. doi: 10.3390/ijms22115650 (PMC8198849; doi:10.3390/ijms22115650)
Supplement: Supplementary file 1 [file ijms-22-05650-s001.zip › Supplementary Tables/Tables S3 to S9 statistics values.doc]

**Table S3: Statistical p values and n values for Figure 1.**

HP: *H. pylori*. ns: not significant.

**Table S4: Statistical p values and n values for Figure 2.**

ns: not significant.

**Table S5: Statistical p values and n values for Figure 3.**

ns: not significant.

**Table S6: Statistical p values and n values for Figure 4.**

ns: not significant.

**Table S7: Statistical p values and n values for Figure 5.**

ns: not significant.

**Table S8: Statistical p values and n values for Figure 6.**

ns: not significant.

**Table S9: Statistical p values and n values for Figure 7.**

ns: not significant.
